# Study on Elastic Helical TDR Sensing Cable for Distributed Deformation Detection

**DOI:** 10.3390/s120709586

**Published:** 2012-07-13

**Authors:** Renyuan Tong, Ming Li, Qing Li

**Affiliations:** 1 School of Information Science & Technology, East China Normal University, No. 500, Dong-Chuan Road, Shanghai 200241, China; E-Mails: tongrenyuan@126.com (R.T.); ming_lihk@yahoo.com (M.L.); 2 College of Mechanical and Electrical Engineering, China Jiliang University, Hangzhou 310018, China

**Keywords:** elastic helical, distributed detection, TDR sensing cable, geological hazard monitoring

## Abstract

In order to detect distributed ground surface deformation, an elastic helical structure Time Domain Reflectometry (TDR) sensing cable is shown in this paper. This special sensing cable consists of three parts: a silicone rubber rope in the center; a couple of parallel wires coiling around the rope; a silicone rubber pipe covering the sensing cable. By analyzing the relationship between the impedance and the structure of the sensing cable, the impedance model shows that the sensing cable impedance will increase when the cable is stretched. This specific characteristic is verified in the cable stretching experiment which is the base of TDR sensing technology. The TDR experiment shows that a positive reflected signal is created at the stretching deformation point on the sensing cable. The results show that the deformation section length and the stretching elongation will both affect the amplitude of the reflected signal. Finally, the deformation locating experiments show that the sensing cable can accurately detect the deformation point position on the sensing cable.

## 1. Introduction

Surface deformation caused by geological hazards is an important phenomenon in geological hazard monitoring, such as landslides. Different kinds of ground deformation sensing technologies have been applied for detecting or measuring surface deformation, such as Global Positioning System (GPS) technology, Brillouin optical time domain reflectometer (BOTDR) and Differential Interferometric Synthetic Aperture Radar (D-InSAR).

The GPS deformation monitoring system has been an important tool for studying surface deformation processes [1–6]. It can provide high-precision (mm level) three-dimensional displacement information of the monitoring points and the monitoring points do not have to be visible. Bai *et al.* built a comprehensive monitoring system including GPS InSAR and inclinometer to study the dynamic deformation process of the Jiaju landslide in Danba (Sichuan, China). With GPS displacement monitoring data, the FLAC^3D^ numerical simulation method was adopted and the stress field, distribution of displacement and plastic zone in the dynamic deformation process were simulated. The simulation results were consistent with monitoring results. GPS deformation monitoring technology is also used in mine area safety monitoring applications. Zhao *et al.* established a systematical GPS monitoring network on the ground surface of the Longshou Opencast Mine in China. After five and half years of monitoring, a 3D numerical model was established to reveal the stress environment around the excavation and opencast slope rock mass, and the rock mass movement and deformation, stress distribution and failure mechanism were discussed. Combined with the monitoring results and field investigation, it pointed out that the overall slope stability is relatively good in the current stage. However, GPS technology can only measure the displacement of monitoring points with GPS observation stations. Rock masses are not rigid bodies. The deformation of different parts of the ground surface is different. One monitoring point's displacement result cannot represent other parts' displacement. In order to monitor ground deformation, the quantity of monitoring points should be large enough. In the Jiaju landslide monitoring system, there are 22 monitoring points. In the Longshou Opencast Mine, the number is nearly 300. In order to monitor so many deformation points, each deformation point has to be measured periodically with a limited amount of GPS equipment. The monitoring period can be several months, which limits the real-time performance of GPS deformation monitoring system.

BOTDR is a kind of distributed deformation monitoring technology [7–11]. It sends a light pulse into an optical fiber fixed along the observed object. According to the relationship between reflected scattered light frequency shift change and optical fiber deformation and time interval between pulsed light and reflected scattered light, BOTDR can locate and measure the deformation point along the optical fiber from the reflected scattered light. Wang and Shi applied BOTDR technology to slope deformation monitoring. However, the optical fiber deformation is very small (generally 15,000 με). If the deformation is too large, the optical fiber will break. This situation has happen in several monitoring systems, such as the slope surface deformation monitoring system in the Guanjia slope of Longli freeway in Zhejiang Province in China, so BOTDR is usually used to measure the deformation of buildings, bridges or dams.

D-InSAR is a wide area surface deformation sensing technology [12–15]. Interferometric Synthetic Aperture Radar takes pictures of the observed object from different view angles at different times. After interferometric processing with these images, D-InSAR technology can give a synoptic view of the deformation events projected along the sensor-target line of sight on areas of hundreds to thousands of square kilometers. The accuracy of D-InSAR can be at cm level or more. Achache *et al.* studied the Saint-Etienne-de-Tinee landslide in the south of France using D-InSAR technology with six interferometry pictures obtained from ERS-1 in 1995 and proved the consistency between the accuracy of D-InSAR technology and the accuracy of other ground monitoring methods. However, image coherence will seriously affect the application of D-InSAR in surface deformation monitoring. Especially at areas with a large amount of vegetation or when a large surface deformation happens in a short time, the coherence may be too low to obtain surface deformation data.

After analyzing ground surface deformation characteristics and the present surface deformation sensing technologies, we put forward a new distributed surface deformation detection technology based on TDR using a special TDR sensing cable. This special TDR sensing cable can overcome BOTDR's intrinsic limit (small deformations). It can detect large distributed deformations in geological hazards.

## 2. TDR Distributed Sensing Technology Background

**Figure 1.**
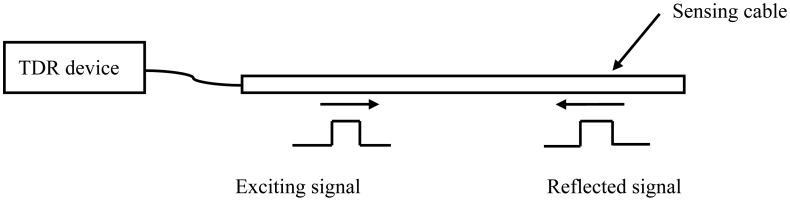
TDR measurement system.

TDR technology is something like radar (Figure 1). A TDR device sends an exciting electrical signal into a TDR sensing cable. The exciting electrical signal can be a short-time pulse or a fast-leading-edge step electrical signal. The electrical signal will be reflected back at the position where the cable impedance is not continuous. This discontinuity can be caused by the change of the environment around the cable or the change of the sensing cable structure. According to the reflected signal waveform, the environmental situation along the cable can be measured and located.

**Figure 2.**
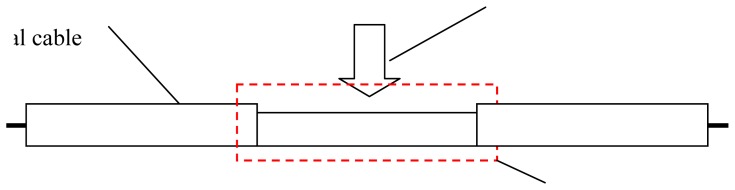
Coaxial cable cross-sectional sharp deformation.

TDR technology has been used in many fields. Using TDR technology, cable fault location equipment can point out where telephone cable is broken or short circuited. It helps workers fix communication networks. TDR is also used in measuring the water content of soils [16,17]. Water content can change soils' dielectric constant, and there is a relationship between soils' dielectric constant and electrical signal's propagation velocity. According to this relationship, Topp measured soil water content with coaxial transmission line sensing cable. Besides water content, TDR technology is also used in underground displacement measurements in landslide monitoring [18–21]. The deformation of soil/rock mass induces the cable's cross-sectional deformation, which then induces the TDR response (Figure 2). Lin measured this deformation with coaxial cable [18].

**Figure 3.**
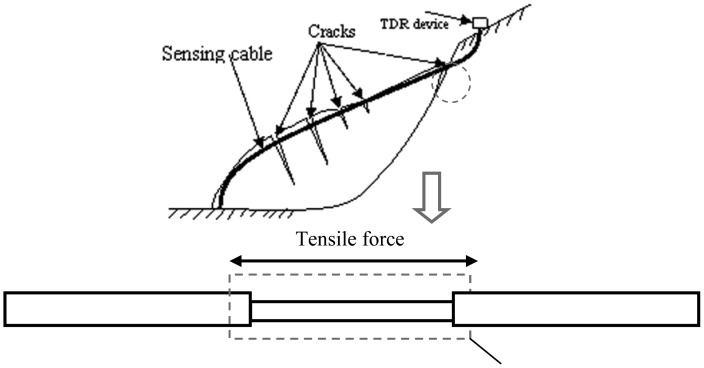
Stretch deformation.

However, traditional TDR sensor cable is hard to apply in surface deformation measurements. Generally, there are two kinds of TDR sensing cables. One is the coaxial cable; the other is the parallel cable (like telephone cable). As Lin did, coaxial cable can measure shear deformation underground, but the TDR sensing cable should have the ability to measure stretch deformation (like BOTDR) in surface deformation monitoring (Figure 3). However, the elasticity of coaxial cable and parallel cable is too small to be used in stretch deformation applications. In order to apply TDR technology in surface deformation distributed detection, an elastic helical TDR sensing cable (EHTSC) is proposed in this paper.

## 3. Elastic Helical TDR Sensing Cable

**Figure 4.**
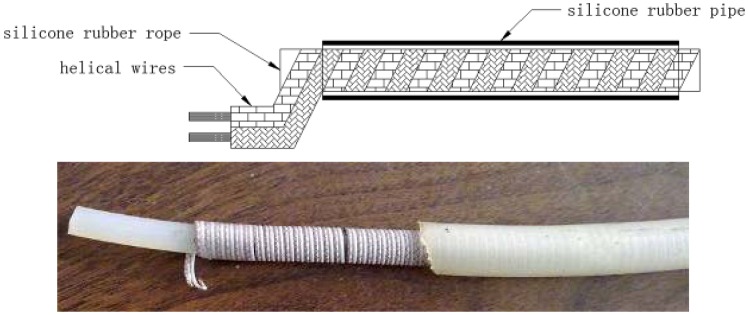
. Structure of elastic helical TDR sensing cable.

As shown in Figure 4, elastic helical TDR sensing cable has a unique structure compared with traditional TDR sensor cable. In the center of the cable is a silicone rubber rope. A couple of parallel wires coil around the rope. We call them helical wires here. There is a silicone rubber pipe around the helical wires. The silicone rubber pipe is used for keeping helical wires away from water in the ground.

**Figure 5.**
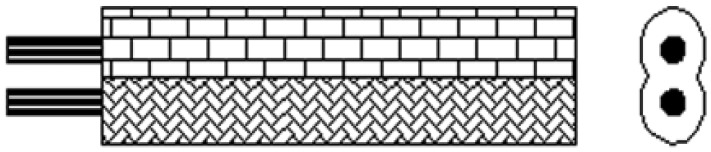
. Parallel wires structure.

**Figure 6.**
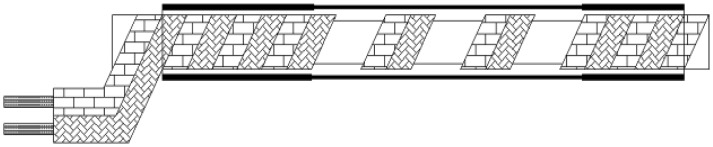
. TDR sensing cable when stretched.

The parallel wires have a plastic sheath as shown in Figure 5, so the distance between two wires in the parallel wires is fixed. Because of its unique structure, EHTSC has two special characteristics. First, it has good elasticity compared with coaxial cable, parallel cable and optical fiber. As shown in Figure 6, when EHTSC is stretched, the helical wires' pitch increases, the silicone rubber rope's diameter becomes smaller and the silicone rubber pipe becomes thinner, but the helical wires are not broken during this process, so EHTSC can have a much bigger deformation range compared with optical fiber and other traditional TDR sensor cables. Second, the EHTSC's impedance will increase when the pitch of the helical wires increases. The distributed deformation detection ability is based on this characteristic. It will be emphatically discussed in this paper. When fixed along a hill's surface, this TDR sensing cable will be stretched if a landslide happens.

## 4. Relationship between Stretching and Impedance

### 4.1. Impedance Analysis

According to the transmission-line theory, transmission-line reflection coefficient is:

(1) $$\rho =U_{r}/U_{t}=(Z_{L}-Z_{C})/(Z_{L}+Z_{C})$$

where *ρ* = Reflection coefficient,
*U_r_* = Reflected signal voltage from TDR sensing cable,
*U_t_* = Exciting signal voltage send by the TDR device,
*Z_L_* = impedance at the deformation point on TDR sensing cable,
*Z_C_* = TDR sensing cable's impedance.

According to Equation (1), the impedance characteristic decides the TDR sensing cable's measurement characteristic. If *Z_L_* > *Z_C_*, a positive reflected signal will be found in the reflected signal waveform. If *Z_L_* < *Z_C_*, a negative signal will be received. Cables with different impedance characteristic can detect different parameters. In order to detect stretching ground surface deformation, a TDR sensing cable's impedance should change when the cable is stretched by ground surface deformation.

**Figure 7.**
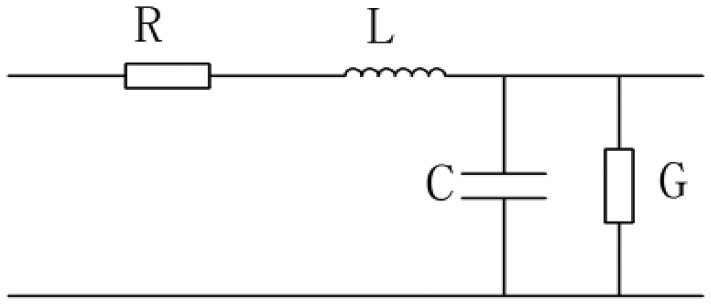
. Transmission-line model.

The TDR sensing cable can be modeled as the circuit shown in Figure 7, where R is the distributed resistance, *L* is the distributed inductance, *C* is the distributed capacitance and *G* is the distributed conductance.

According to the transmission-line model, the impedance model of the TDR measurement cable is:

(2) $$Z_{C}=\sqrt{\left(R+j\omega L)/(G+j\omega C\right)}$$

where *ω* = frequency of the signal and *j* = complex unit.

If *ω* is large enough, *R* and *G* can be ignored compared with *jωL* and *jωC*. The impedance can be changed to Equation (3):

(3) $$Z_{C}=\sqrt{L/C}$$

Compared with coaxial cable and parallel cable, the electromagnetic fields of helical wires is much more complicatedand it is difficult to calculate L and C precisely. To simplify the analysis, we assume that the signal frequency is high enough and the electromagnetic wave along the helical structure is approximately a plane wave. Based on this assumption, the electromagnetic fields can be analyzed using static electromagnetic analysis methods.

**Figure 8.**
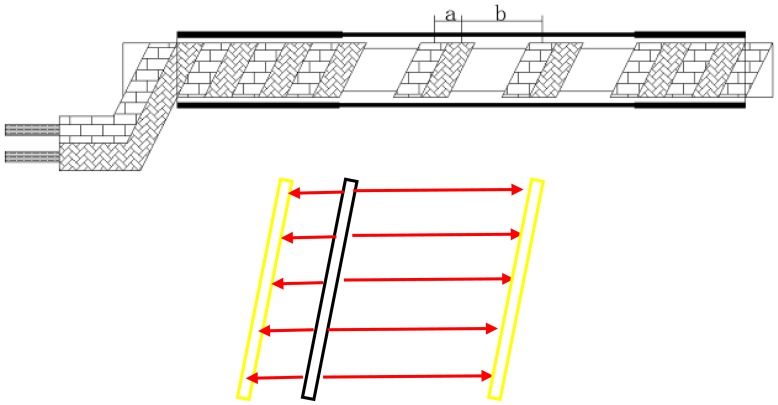
. Electric field distribution.

Figure 8 shows the surface electric field distribution of the helical wires. *E_a_* is the internal electric field of parallel wires. Besides the internal electric field *E_a_*, there is an electric field *E_b_* between adjacent helical wires as the helical wires are closely entwined.

According to the surface electric field distribution, the distributed capacitance C consists of two parts:

(4) $$C=C_{a}+C_{b}$$

where *C_a_* = internal distributed capacitance in parallel wires,
*C_b_* = distributed capacitance between adjacent helical wires.

If the signal frequency is high enough, most of charge will be on the surface of the wires. According to the parallel wires capacitance calculation method, the distributed capacitance can be written as:

(5) $$C=\pi ɛ(\frac{1}{\ln\frac{d_{a}}{r}}+\frac{1}{\ln\frac{d_{b}}{r}})=\pi ɛ\frac{\ln\frac{d_{a}d_{b}}{r^{2}}}{\ln\frac{d_{a}}{r}\ln\frac{d_{b}}{r}}$$

where *r* = wire radius,
*d_a_* = distance of space a,
*d_b_* = distance of space b,
*ε* = permittivity.
*r* is much smaller than *d_a_* and *d_b_* here.

When the signal frequency is high enough, there is a relationship between distributed capacitance and distributed inductance:

(6) $$v=\frac{1}{\sqrt{LC}}=\frac{1}{\sqrt{ɛ\mu }}$$

where *v* = propagation velocity and *μ* = permeability.

**Figure 9.**
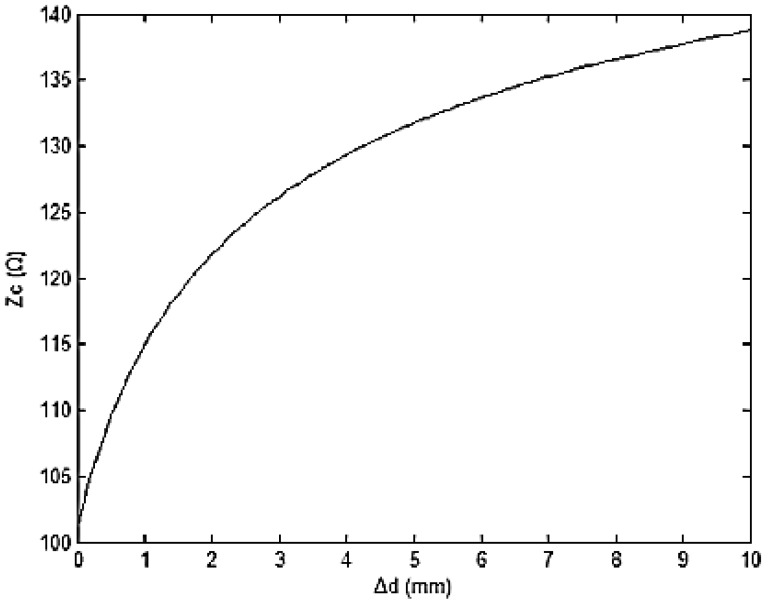
Relationship curve between *Δd* and *Z_C_*.

In the initial state, *d_a_* = *d_b_* = *d*. According to Equations (3), (5) and (6), EHTSC's impedance can be written as:

(7) $$Z_{C}=\frac{1}{\pi }\sqrt{\frac{\mu }{ɛ}}\frac{\ln\frac{d}{r}\ln\frac{d+\Delta d}{r}}{\ln\frac{d(d+\Delta d)}{r^{2}}}$$

where *Δd* is the change value of *d_b_*. If the relative permeability is 1, the relative permittivity is 1.2, wires radius *r* is 0.2 mm and initial distance *d* is 1.27 mm. Figure 9 is the relationship curve between *Δd* and *Z_C_*.

The relationship curve shows that EHTSC's impedance increases when the cable is stretched and the rate decreases when *Δd* increases. That means EHTSC can effectively detect the deformation in a certain range near *Δd* = 0.

### 4.2. Impedance Experiment

**Figure 10.**
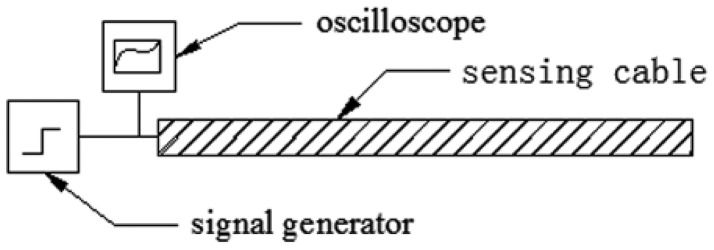
Impedance experiment.

Until now the relationship between elongation and impedance has been discussed through transmission-line theory. An impedance experiment is done here to verify the theoretical analysis. Figure 10 is the illustration of the impedance test.

The impedance experiment system consists of three parts, namely a RIGOL DG3121A signal generator, Agilent MSO7054A oscilloscope and elastic helical TDR sensing cable. The RIGOL DG3121A can generate a fast-leading-edge (less than 2 ns) electrical step signal. Its output impedance is 50 Ω. The bandwidth of the Agilent MSO7054A is 500 MHz and its sampling frequency is 2 GHz. The signal generator and oscilloscope compose a TDR device. The sensing cable's initial length is 270 mm. Its diameter is about 10 mm.

**Figure 11.**
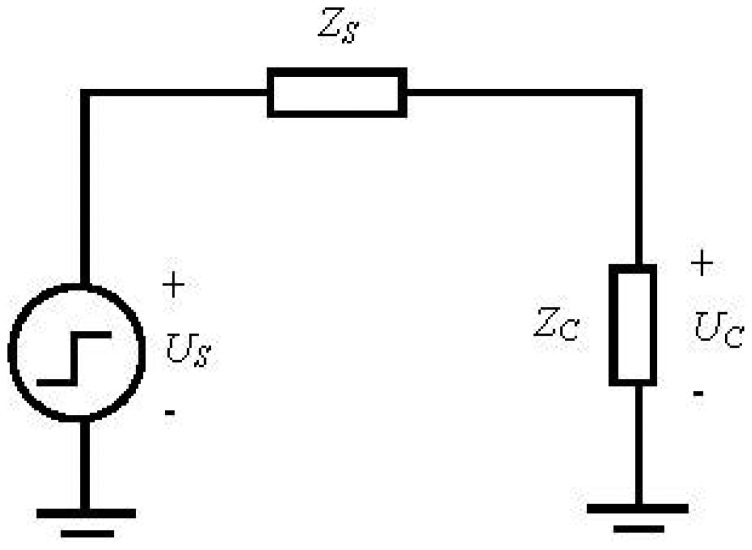
Transient circuit model.

Figure 11 shows the transient circuit model of the impedance experiment. *Z_C_* is the impedance of EHTSC. *Zs* is signal generator's output impedance. Equation (8) is the relationship between the step signal voltage *U_S_* and the transient voltage *U_C_* on *Z_C_*:

(8) $$U_{c}=\frac{Z_{c}}{Z_{s}+Z_{c}}U_{s}$$

**Figure 12.**
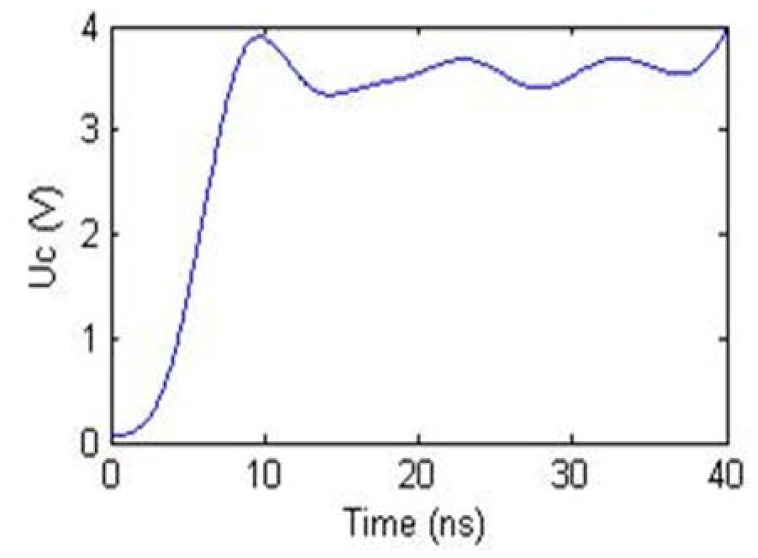
The transient voltage waveform under step excitation signal.

Figure 12 shows the transient voltage waveform in the EHTSC. The step signal voltage *U_S_* created by the signal generator is 5 V. Because of the sensing cable impedance, the transient voltage is lower than the step signal voltage. However, the waveform is not a straight line. The change of the transient voltage can be caused by the non-uniformity of the cable's impedance. In order to find the relationship between the transient voltage and the elongation of the sensing cable, the average transient voltage is calculated.

**Figure 13.**
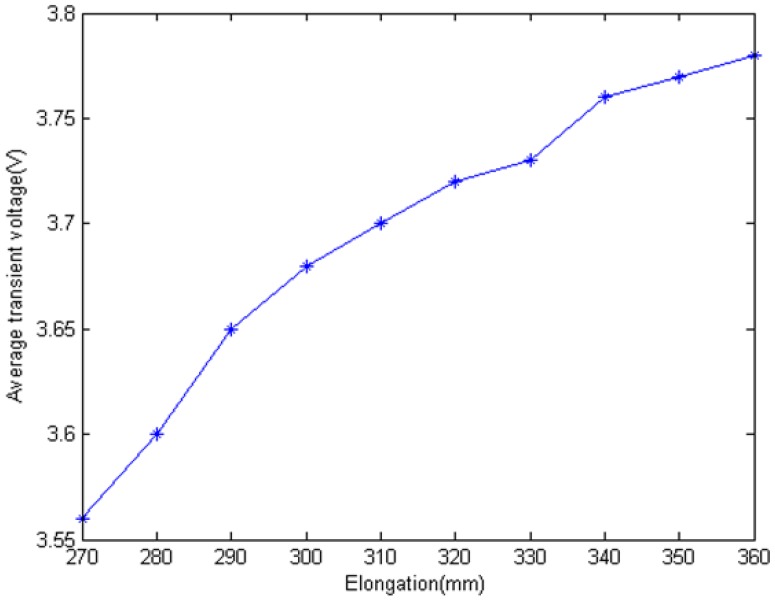
Relationship between elongation and average transient voltage.

**Figure 14.**
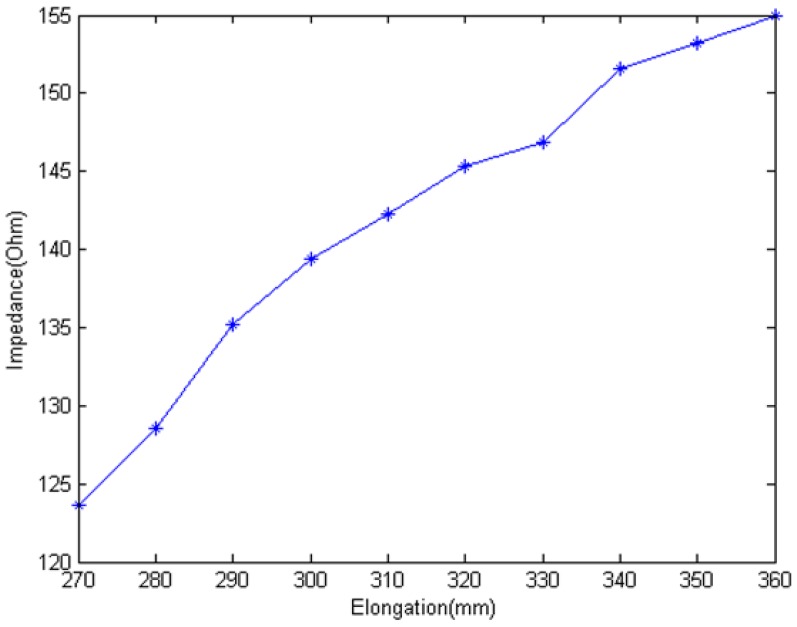
Relationship between impedance and elongation.

Figure 13 shows that the transient voltage *U_C_* increases when EHTSC is stretched. And the rate decreases with the elongation increasing. According to Equation (8), we can calculate the impedance of EHTSC as Figure 14 shows.

The experiment results verify our conclusion that the impedance of EHTSC increases when it is stretched. There is a difference between Figures 9 and 14. This difference may be caused by the connector and the non-uniform structure of the manual sensing cable.

## 5. Research on Local Deformation

**Figure 15.**
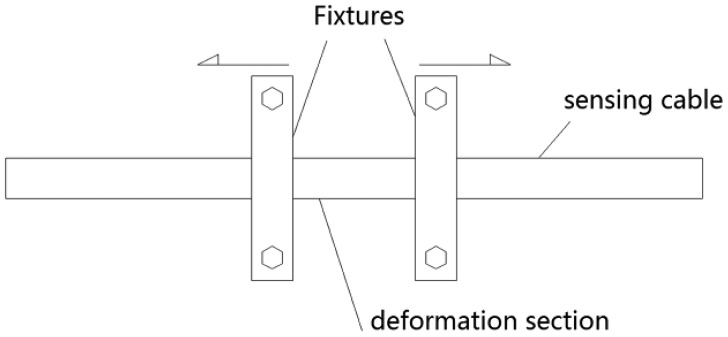
Illustration of the local deformation TDR experiment.

The last section shows that the impedance of an elastic helical structure TDR sensing cable increases when it is stretched. As Equation (1) shows, this is the physical basis for TDR technology. According to transmission line theory, a positive signal will be reflected if the local impedance increases. Figure 15 shows the method of the local deformation experiment. The TDR device is composed of the signal generator and the oscilloscope which have been mentioned above. We choose a deformation section on the sensing cable. Two fixtures are fixed at the ends of the deformation section. When we push the fixtures away from each other a local deformation is created on the deformation section. We use a Vernier caliper to measure the distance change between the two fixtures as the deformation variation.

**Figure 16.**
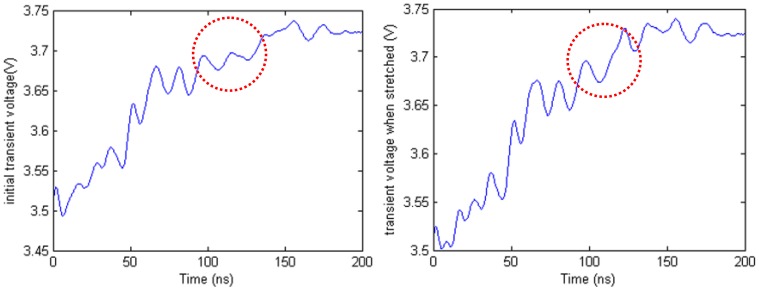
Comparison transient voltage waveforms.

The length of the deformation section is 25.0 mm. Then the deformation section is stretched to 54.6 mm. Figure 16 shows the transient voltage in the initial state and the transient voltage when the cable is stretched. From the waveform in the red circle, we can see that there is a difference between the two waveforms. Compared with non-uniformity influence, the waveform difference caused by stretching is very small. To obtain deformation directly from the waveform is difficult.

**Figure 17.**
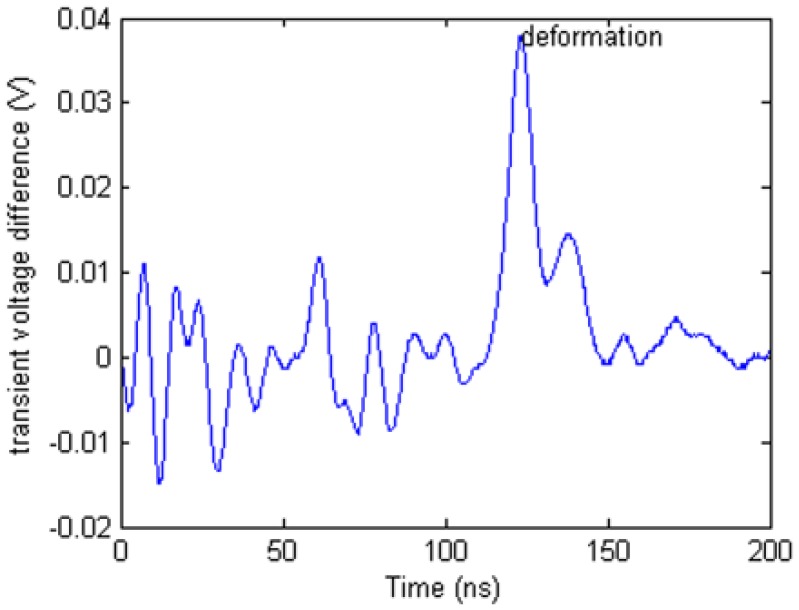
. Difference waveform.

To obtain the local change of the transient voltage, a comparison method is applied in the TDR data processing. The difference waveform in Figure 17 is created by calculating the stretched waveform minus the initial state waveform. There is an obvious spike in the waveform which is ceased by the stretching deformation. Beside the highest spike, there are many other lower spikes. Those spikes can be created by the TDR device measurement uncertainty.

**Figure 18.**
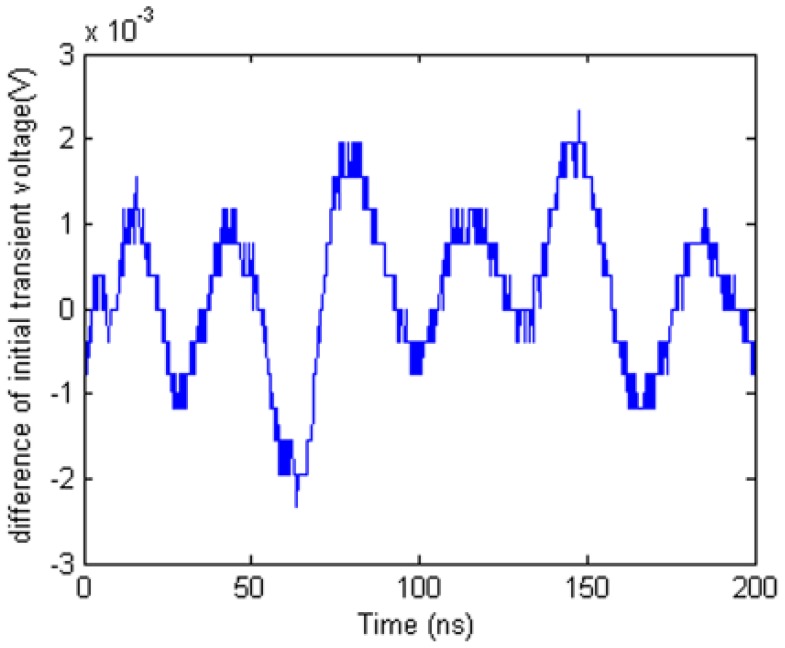
Difference waveform between two initial transient voltage waveforms.

Figure 18 shows that there is a difference between two initial transient voltage waveforms. It means that the uncertainty of TDR device will affect the difference waveform whether deformation is happened.

**Figure 19.**
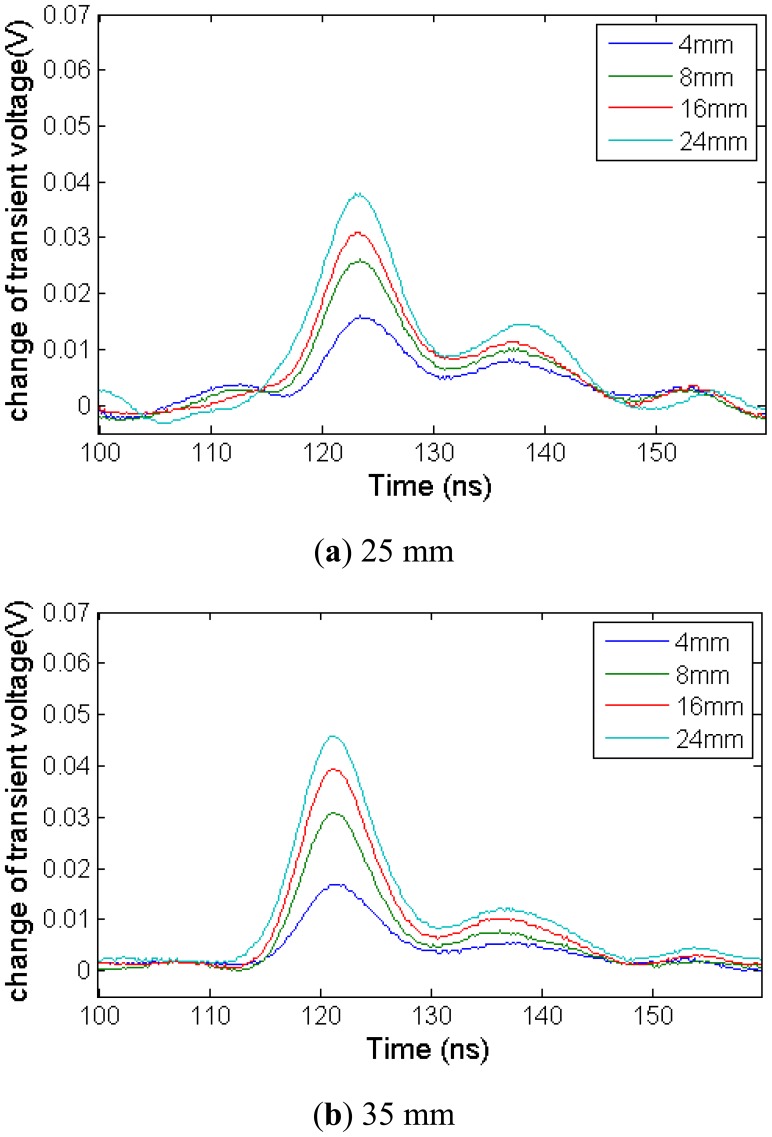

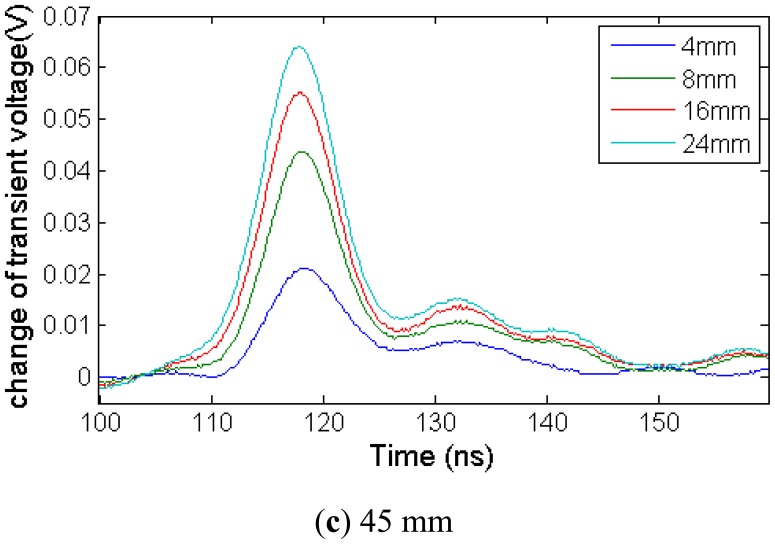
Change of transient voltage waveform with different deformation section lengths.

In order to detect ground surface deformation, the relationship between reflected waveform spike and stretching deformation is researched. In this experiment, we select three different deformation sections—25 mm, 35 mm, 45 mm. Figure 19 shows the local deformation TDR experiment results with the comparison method.

With different deformation section, the waveforms show that the spike magnitude increase when the sensing cable is stretched from 4 mm to 24 mm with different deformation section lengths. It verifies the conclusion which is made according to the theoretical analysis above.

**Figure 20.**
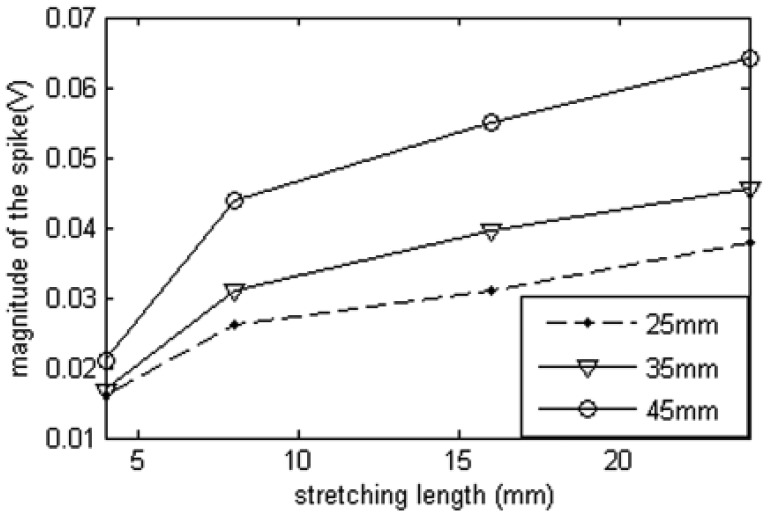
Relationship between deformation section lengths and magnitudes of the spikes.

However, the reflected waveform spike magnitude will be affected by the length of deformation section. From Figure 20, we can see that the spike magnitude for the longer deformation section is higher than the others with short deformation sections for the same stretching length. Therefore, deformation length cannot be calculated simply from the spike magnitude.

This problem had been put forward by Lin in quantification of coaxial cable deformation with TDR [18,22]. He solved this problem with a non-uniform transmission line multisection model. However, his method is hard to apply to this experiment. EHTSC used in this experiment is much more non-uniform than commercial coaxial cable which was used by Lin in the localized shear deformation. In our opinion, this problem may be solved by improving the EHTSC manufacturing process.

## 6. Locating Deformation Point Test

**Figure 21.**
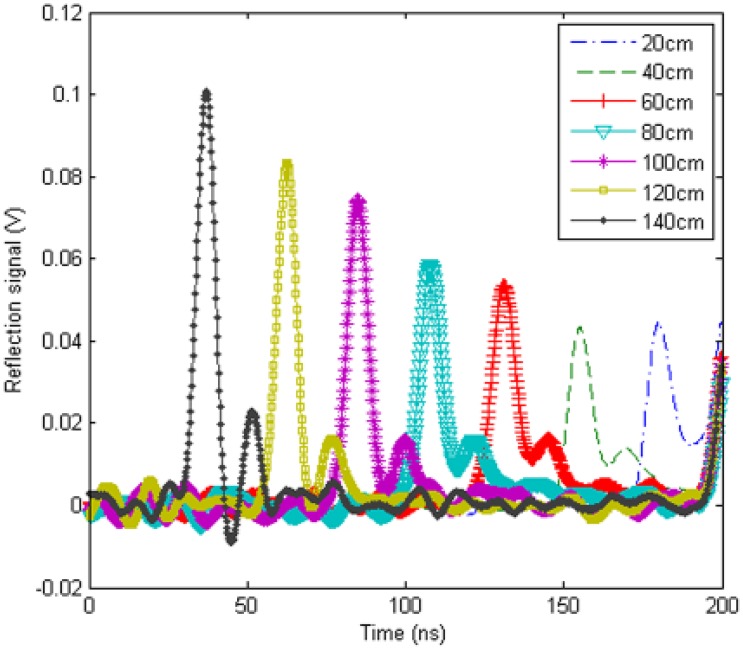
Reflection signal waveforms when the deformation happens at different positions on the sensing cable.

Although further research should be done in calculating out the cable's stretching length, EHTSC's ability to locate stretching deformation points is a useful characteristic. Because of the non-uniformity of the cable, the location of deformation points still uses the comparison method to deal with TDR data. Figure 21 shows the reflection waveforms when the deformation happens at different positions.

Total length of the sensing cable in the experiment is 2 m. The deformation section length is 30 mm. The stretching length is 20 mm, which means the deformation section length changes from 30 mm to 50 mm. The distances shown in Figure 21 are from the terminal of the sensing cable to the deformation section. The waveforms indicate that the magnitudes of the spikes decrease when the deformation positions move away from the TDR device. It can be caused by multiple reflections and conductive loss. The multiple reflections caused by the non-uniformity of the cable decrease the spike's magnitude. Therefore, when we study on the relationship between the reflected spike and the stretching length, the distance influence should be included.

**Figure 22.**
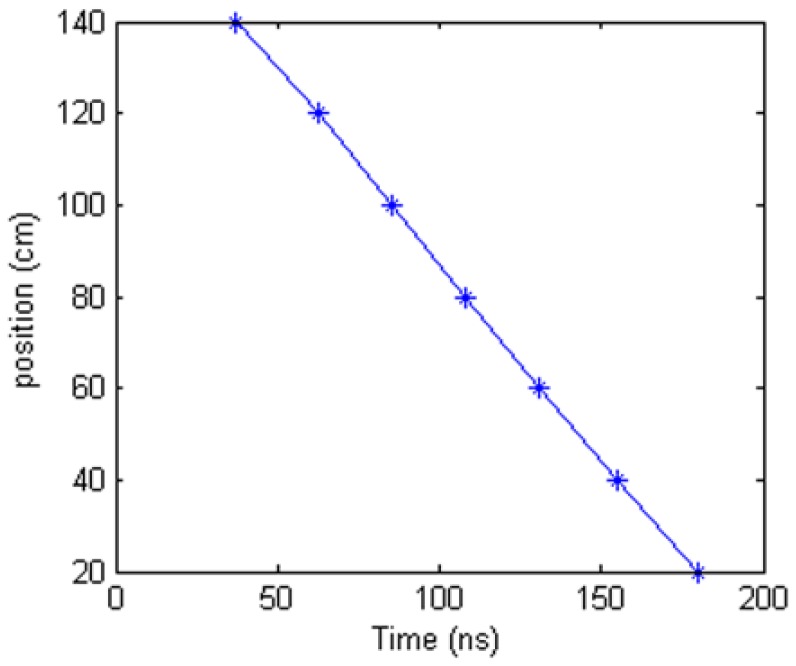
Result after demarcation.

Figure 22 is the relationship between spike positions located by the oscilloscope from Figure 21 and the real deformation positions. It is a linear relationship. If choosing a straight line from the point at 20 cm to the point at 140 cm to fit the experiment results in Figure 22, the max location error is 1.6 cm. Using this method, the deformation points can be located by EHTSC.

## 7. Conclusions

This research describes an elastic helical TDR sensing cable which is suitable for distributed ground surface deformation detection. From the research above, we can draw some conclusions as follows:

1. There is a relationship between an elastic helical TDR sensing cable's structure and its impedance. This relationship is analyzed based on transmission-line model and shown as Equation (7). From Equation (7) we know that the cable impedance increases when the cable is stretched. This characteristic is verified by experiments.
2. A positive pulse reflected signal will be generated at the deformation point on the sensing cable. Because of non-uniformity of the cable structure, it is difficult to obtain the reflected signal caused by deformation. To overcome this problem, we use a comparison method to deal with the reflected signal wave and get a clear positive pulse reflected signal from the noise.
3. The reflected signal amplitude has a relationship with the deformation section length, stretching elongation and distance from TDR device. From experiments, we can see that the longer the deformation section length, the longer the stretching elongation and a short distance from TDR device will give a higher reflected signal amplitude.
4. The sensing cable can locate the deformation point accurately. After demarcation, the elastic helical TDR sensing cable could effectively locate the position of the deformation point in the simulation ground surface deformation experiment. In further application, the sensing cable will be fixed on the surface of a landslide. When the landslide happens, the sensing cable will be stretched. Through the sensing cable, we can monitor any deformation on the landslide along the sensing cable.
